# Supplementary material for: Pharmacognostic Study, Diuretic Activity and Acute Oral Toxicity of the Leaves of Xiphidium caeruleum Aubl. Collected in Two Different Phenological Stages
Source: Plants (Basel). 2023 Mar 10;12(6):1268. doi: 10.3390/plants12061268 (PMC10052520; doi:10.3390/plants12061268)

Figure S1. Thin Layer Chromatography profile of the aqueous extract of the leaves of X. caeruleum collected in the vegetative and flowering stage. Observations were done under UV light at 254 nm (A) and 366 nm (B) and after revealing with anisaldehyde / heat (C). Spots are indicated by parenthesis.

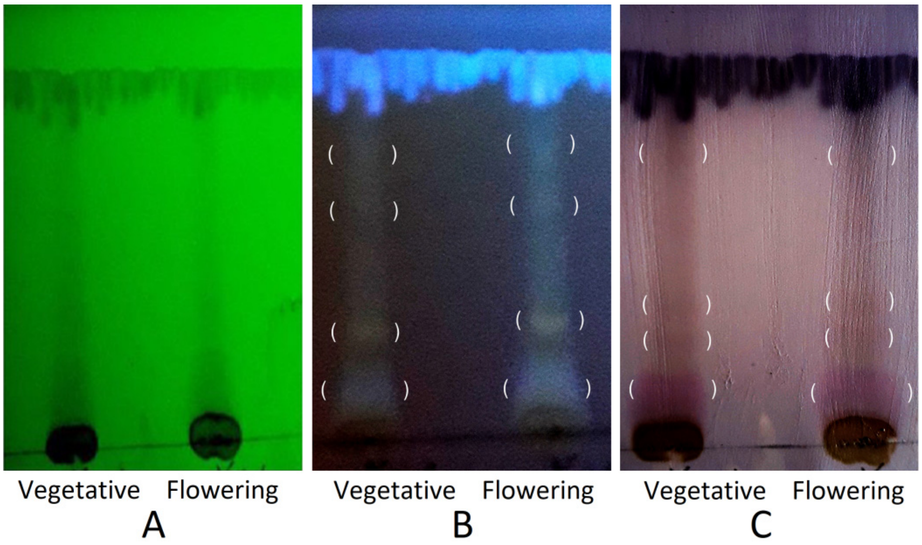

Figure S2. Ultraviolet (A) and Infrared (B) profiles of the aqueous extract of the leaves of X. caeruleum collected in the vegetative and flowering stage of the plant.

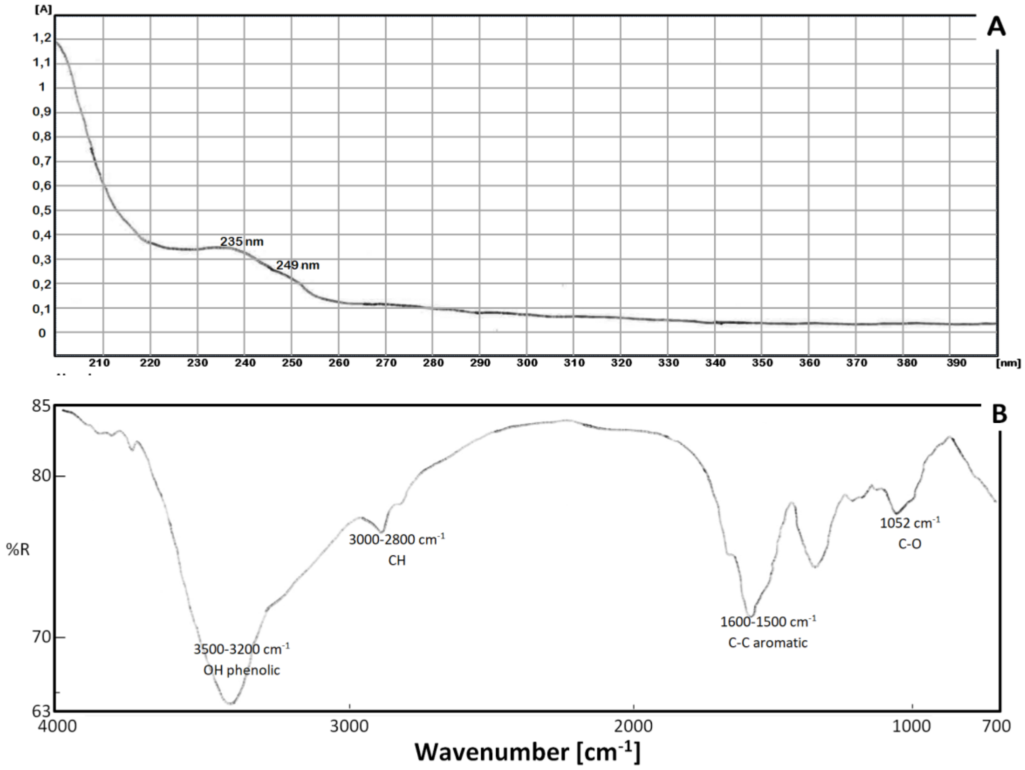

Figure S3. Calibration curve of Gallic acid.

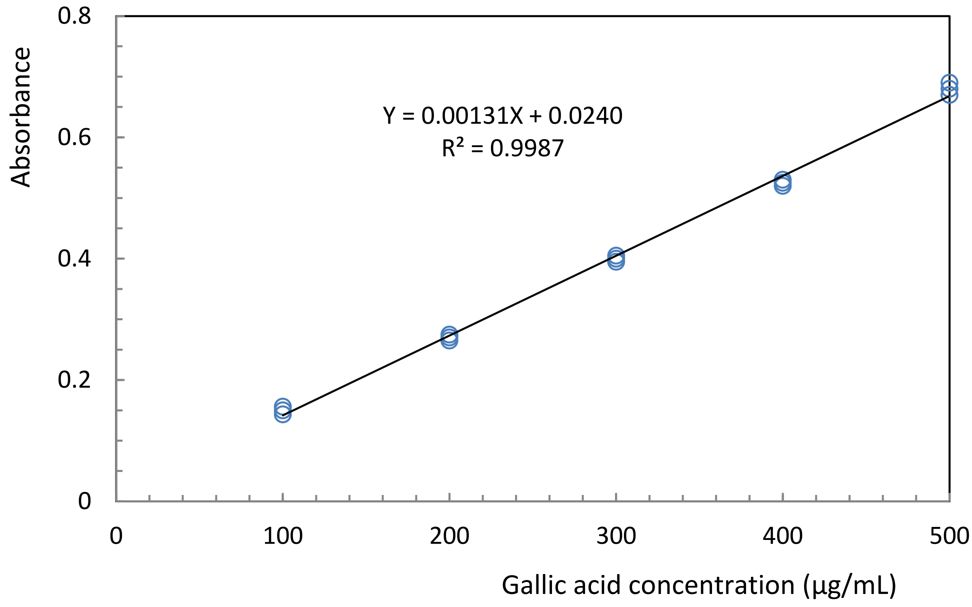

Figure S4. Calibration curve of Quercetin.

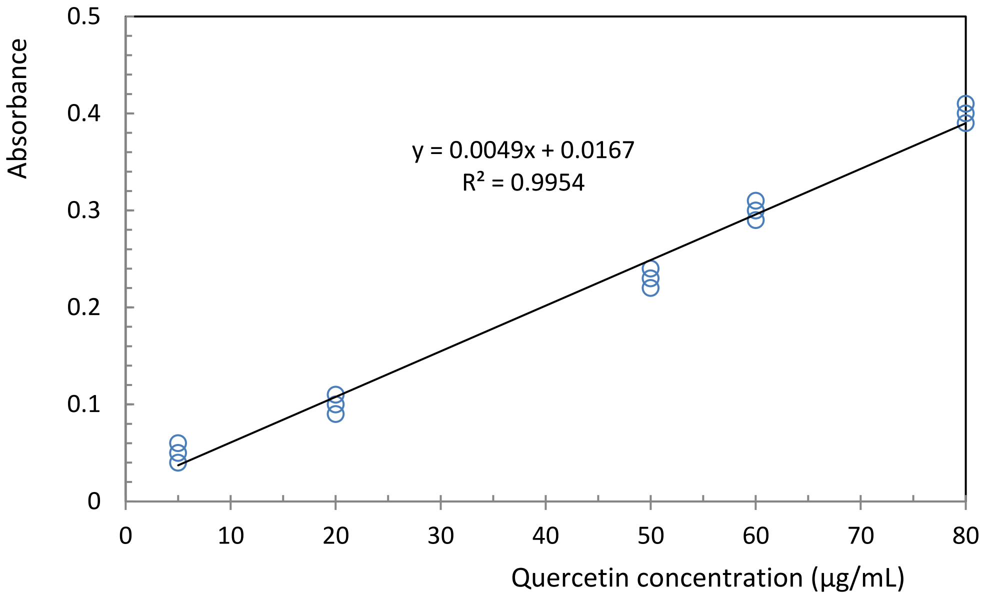

Supplement: Supplementary file 1 [file plants-12-01268-s001.zip › plants-2197561-supplementary.pdf]
